# Chromosome structure dynamics during the cell cycle: a structure to fit every phase

**DOI:** 10.15252/embj.201798014

**Published:** 2017-09-04

**Authors:** Christopher Barrington, Dubravka Pezic, Suzana Hadjur

**Affiliations:** ^1^ Research Department of Cancer Biology Cancer Institute University College London London UK

**Keywords:** Cell Cycle, Chromatin, Epigenetics, Genomics & Functional Genomics, DNA Replication, Repair & Recombination

## Abstract

Chromosomes undergo dramatic morphological changes as cells advance through the cell cycle. Using powerful molecular and computational methods, several recent studies revealed an outstanding complexity of continuous structural changes accompanying cell cycle progression. In agreement with cell division being a fundamental cellular process, characteristic features of cell cycle stage‐specific genome structure are conserved from yeast to mouse. These studies further shine light on the critical roles that SMC complexes, already well known as fundamental regulators of chromosome topology, have in orchestrating structural dynamics throughout the cell cycle.

Molecular methods such as Hi‐C measure physical contacts between DNA fragments in an unbiased and genome‐wide manner (Lieberman Aiden *et al*, 2009), permitting researchers to describe the higher‐order folding principles of chromosomes with great resolution and in a high throughput manner (Dixon *et al*, 2012; Nora *et al*, 2012; Sexton *et al*, 2012). Four recent studies have harnessed the power of Hi‐C and its statistical analyses to further our understanding of the dramatic structural changes that occur within chromosomes during cell cycle progression. Collectively, the work in *Schizosaccharomyces pombe* (Kakui *et al*, 2017), *Saccharomyces cerevisiae* (Lazar‐Stefanita *et al*, 2017; Schalbetter *et al*, 2017) and mouse ES cells (Nagano *et al*, 2017) has revealed distinct cell cycle stage chromosome structures, the importance of structural maintenance of chromosome (SMC) proteins throughout this process and the conservation of structural features between species.

Chromosome structure during the cell cycle has been studied in several independent laboratories using multiple synchronisation methods and in diverse eukaryotic models. Together, the studies reinforce previous work (Naumova *et al*, 2013) that specific stages of the cell cycle can be characterised by a distinct contact composition, and highlight the conservation of chromosome organisation associated with specific cell cycle stages. Transcription‐compatible G1 chromatin is characterised by a higher probability of short‐range intra‐chromosomal contacts compared to long‐range contacts. The extent of short contacts differs between organisms depending on the genome and chromosome size. During DNA replication, there is an enrichment of long‐range intra‐chromosomal contacts with respect to short‐range. Interestingly, cells in G2 and mitosis exhibit a further specific increase in short‐range contacts identified by Hi‐C, indicative of the gradual axial compaction and individualisation of chromosomes required for cell division (Fig 1A).

**Figure 1:**
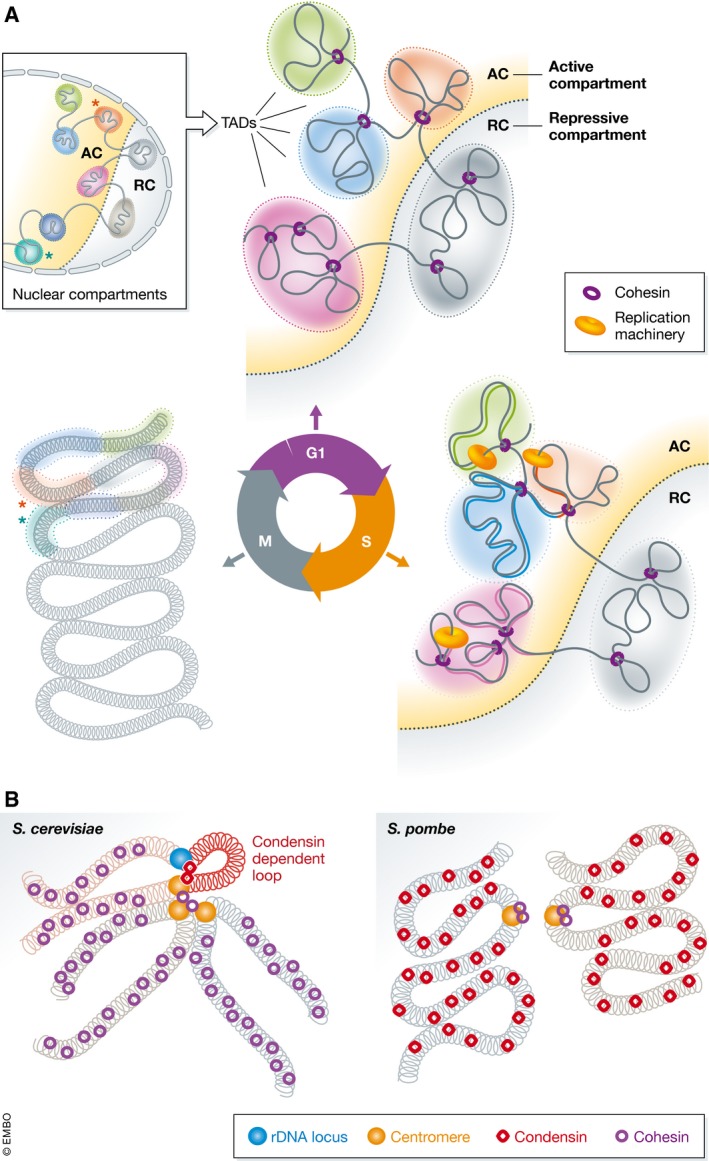
Chromosome structures and SMC proteins during the cell cycle (A) Schematic representations of chromosome structure during the cell cycle. TADs on a section of a chromosome are indicated as shaded areas in active (AC) or repressive (RC) compartments, separated by the dotted line. Cohesin is shown in purple and replication machinery in orange on the DNA. In G1, TADs are insulated from one another and occupy distinct nuclear space and compartments. During S‐phase, DNA is replicated at specific times, from early to late replicating domains indicated by proportion of replicated DNA in the TAD. TAD insulation is maintained, albeit to a lesser extent, but compartmentalisation increases. Once in M‐phase, the chromatin is highly compacted with TAD structure barely identifiable and abundant very‐long‐range contacts emerge between distant TADs (e.g. compare the relationship between the orange and turquoise TADs marked with stars in the zoom‐out of G1‐phase to the M‐phase). (B) Comparison of the structure of mitotic chromosomes in yeast (for simplicity, only individual sister chromatids are shown). *Saccharomyces cerevisiae* chromosome arms are compacted by cohesin compared to *Schizosaccharomyces pombe* where condensin is required. The rDNA locus of *S. cerevisiae* is brought into proximity of the centromere by condensin, which is not required at other loci.

The advancement of single cell sequencing methodologies has highlighted the cell‐to‐cell variability inherent in populations of cells that a traditional analysis would aggregate. A significant contributor to this variability could be the dynamic changes in genome structure that underlie the cell cycle, a source of variation that cannot be fully accounted for by synchronisation of populations or genetic mutation alone. Nagano *et al* (2017) sought to quantify the cell‐to‐cell variability during the cell cycle by adapting Hi‐C for single cell analysis. By combining mitotic contact frequency signatures with a “replication score” for each cell the authors were able to rank the single‐cell Hi‐C datasets by their cell cycle progression. The analysis reaffirmed the prevalence of local contacts during interphase and the enrichment of long‐range mitotic contacts during mitosis and early G1. Importantly, as the data were collected from single cells, the authors were able to reveal that the composition of genome structure is dynamic throughout the cell cycle. This progressive conformational change from local to mitotic contacts indicated that cells are in a constant state of conformational flux throughout their lifetime. Such continuous structural reorganisation was also observed by Lazar‐Stefanita *et al* (2017) in yeast populations synchronised at specific cell cycle checkpoints.

Nagano *et al* (2017) showed that CTCF loops, topologically associated domain (TAD) insulation and compartmentalisation can be observed throughout the cell cycle. While TAD insulation is observed throughout S‐phase, it is reduced when coupled to the replication process. In contrast, compartmentalisation had the opposite trajectory, whereby it increases throughout G1‐ and S‐phase. Notably, insulation, compartmentalisation and CTCF loops (which are likely stabilised by cohesin) are lost during mitosis, when the chromatin is most compact (Fig 1A). However, using Hi‐C it cannot be determined whether the CTCF/cohesin contact is removed or becomes hidden beneath the extensive compaction.

The role of SMC complexes, cohesin and condensin, in dynamic genome restructuring during the cell cycle was addressed in *S. pombe* (Kakui *et al*, 2017) and *S. cerevisiae* (Lazar‐Stefanita *et al*, 2017; Schalbetter *et al*, 2017). Both yeasts have one cohesin and one condensin complex. Their genomes are comparable in size (14 and 12 Mb, respectively), but have different organisations. While the genome of *S. cerevisiae* is divided between 12 chromosomes, whereas *S. pombe* has three. The authors combined genetic ablation with Hi‐C analysis of genome structure on populations of cells from individual cell cycle phases, taking advantage of genetic and chemical methods to arrest the cells at particular stages.

While the cell cycle‐specific structures observed depended on SMC complexes, the roles of cohesin and condensin seem to be different in different organisms (Fig 1B, right). Both Schalbetter *et al* (2017) and Lazar‐Stefanita *et al* (2017) show that the increase in centromere clustering which occurs as cells progress from G1 into mitosis in *S. cerevisiae* depends on both condensin and cohesin. In contrast, cohesin but not condensin is crucial for gradual compaction of sister chromatids and the mitotic structure of the chromosomal arms. The increase in long‐range intra‐chromosomal contacts concomitant with DNA replication depends on cohesin. Condensin is in turn crucial for structuring the rDNA locus. Earlier studies have shown that condensin accumulates on the rDNA array, which occupies ~1.8 Mb of the small *S. cerevisiae* genome, and plays a role in maintenance of the rDNA copy number and correct segregation of the locus.

Kakui *et al* (2017) describe the dependency of structural changes in *S. pombe* genome throughout the cell cycle on condensin (Fig 1B, left). They show that reorganisation of interphase chromatin (characterised by many small domains), into the mitotic form (characterised by smaller number of larger domains), occurs in the presence of condensin. This process increases rigidity of chromatin, and in the absence of condensin, mitotic chromosomes show much greater mobility compared to wild‐type cells.

These studies showcase, on one hand, the deeply conserved principles of structural changes of the genome and overall behaviour of chromosome structure through cell cycle phases and, on the other hand, flexibility in the mechanisms that lead to these structures. Budding yeast has 12 smaller chromosomes, and a mitotic spindle present throughout the cell cycle. Cytologically, its chromosomes condense little; mitosis starts very early, almost overlapping with S‐phase which leaves G2‐phase barely distinguishable. In contrast, fission yeast has three larger chromosomes and a cell cycle more similar to higher eukaryotes. Powerful modern chromosome structure‐probing Hi‐C methodology reveals now that the chromosomes of the two yeasts condense in much the same way, while the two SMC complexes acquire species‐specific functions in chromosome compaction. By employing similar high‐throughput approaches, future experiments will no doubt address how the different SMC complexes work together in higher organisms to orchestrate the crucial structures required for cell cycle progression.
